# Beyond amyloid and tau: rethinking Alzheimer’s disease through less explored avenues

**DOI:** 10.1098/rsob.240035

**Published:** 2024-06-12

**Authors:** M. Gyimesi, R. K. Okolicsanyi, L. M. Haupt

**Affiliations:** ^1^ Stem Cell and Neurogenesis Group, Genomics Research Centre, Centre for Genomics and Personalised Health, School of Biomedical Sciences, Queensland University of Technology (QUT), 60 Musk Ave, Kelvin Grove, Queensland 4059, Australia; ^2^ Max Planck Queensland Centre for the Materials Sciences of Extracellular Matrices, Brisbane, QLD 4059, Australia; ^3^ Centre for Biomedical Technologies, Queensland University of Technology (QUT), 60 Musk Ave, Kelvin Grove, Queensland 4059, Australia; ^4^ ARC Training Centre for Cell and Tissue Engineering Technologies, Brisbane, QLD 4059, Australia

**Keywords:** Alzheimer’s disease, Notch signalling pathway, heparan sulfate proteoglycans, adult hippocampal neurogenesis

## Abstract

Neurodegenerative diseases, particularly Alzheimer’s disease (AD), pose a significant challenge in ageing populations. Our current understanding indicates that the onset of toxic amyloid and tau protein pathologies initiates disease progression. However, existing treatments targeting these hallmark symptoms offer symptomatic relief without halting disease advancement. This review offers an alternative perspective on AD, centring on impaired adult hippocampal neurogenesis (AHN) as a potential early aetiological factor. By delving into the intricate molecular events during the initial stages of AD (Braak Stages I–III), a novel hypothesis is presented, interweaving the roles of Notch signalling and heparan sulfate proteoglycans (HSPGs) in compromised AHN. While acknowledging the significance of the amyloid and tau hypotheses, it calls for further exploration beyond these paradigms, suggesting the potential of altered HS sulfation patterns in AD initiation. Future directions propose more detailed investigations into early HS aggregation, aberrant sulfation patterns and examination of their temporal relationship with tau hyperphosphorylation. In challenging the conventional ‘triggers’ of AD and urging their reconsideration as symptoms, this review advocates an alternative approach to understanding this disease, offering new avenues of investigation into the intricacies of AD pathogenesis.

## 1. Background

Neurodegenerative diseases, particularly dementias, present a formidable challenge as our ageing population continues to rise. Among these conditions, Alzheimer’s disease (AD) stands as a prevalent force, accounting for 60–80% of diagnosed dementia cases [1]. AD is most commonly seen in the form of late-onset sporadic AD (LOAD) impacting individuals aged 65 and above, with the primary genetic risk factor identified as Apolipoprotein E allele ε4 (*APOE* ε4) [2]. Approximately 5–6% of AD cases are classified as early-onset AD (EOAD), occurring before the age of 65, with only 0.6% of these exhibiting the causative, autosomal dominant genetic mutations in the Presenilin (*PSEN1* and *PSEN2*) and Amyloid Precursor Protein (*APP*) genes [3,4]. Although these subtypes exhibit nuanced differences in neuropathology and psychology, they share commonality via the presence of the hallmark amyloid and tau protein pathologies [5]. Unfortunately, whether these factors serve as symptoms or represent the cause of AD remains elusive, sparking ongoing debate among researchers. Despite the wealth of new insights into the dysregulated processes underlying the appearance of toxic amyloid plaques and hyperphosphorylated tau protein, treatments targeting these continue to fail to cure AD, and offer only minimal symptomatic relief [6–10].

This raises a pivotal question: have we thoroughly explored the classic amyloid and tau hypotheses with no causative mechanism identified, signalling a need for a paradigm shift? Furthermore, the current affinity among researchers to view new evidence solely through the lens of the well-established amyloid and tau hypotheses could be hindering the exploration of other genes and proteins and their multifaceted roles within the human brain as potential initiators and drivers of AD pathology. Perhaps it is time to consider a novel perspective on AD, emphasizing impaired neurogenesis as an early aetiological factor. In this review, we explore the existing knowledge of adult hippocampal neurogenesis (AHN) and extend our inquiry into the perspective that compromised AHN could serve as a fundamental player in the prodromal and preclinical phases of AD, even preceding the amyloid and tau features. We aim to unravel the molecular interplay underlying impaired AHN, thus contributing to a deeper understanding of the complex landscape of AD pathogenesis.

### 1.1. Healthy neurogenesis

**Figure 1.**
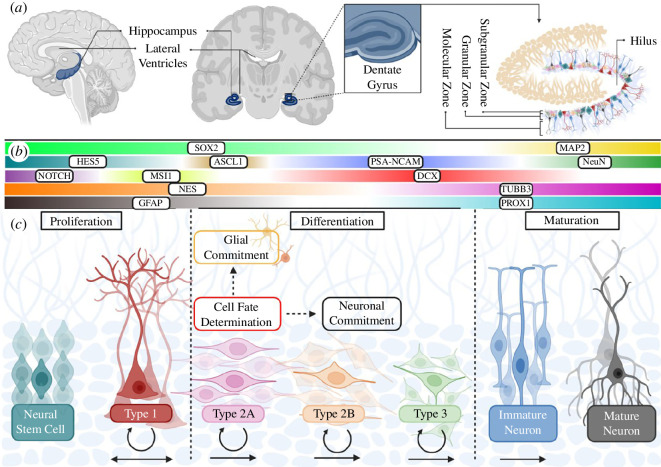
Adult hippocampal neurogenesis (AHN). This illustration depicts (*a*) the anatomical context of the hippocampus within the adult human brain, emphasizing the region known as the dentate gyrus, where AHN unfolds, particularly in the sub-granular zone. (*b*) The diagram depicts changes in neural marker expression at each stage of AHN, encompassing both protein and gene levels. (*c*) The diagram outlines the sequential phases of AHN, elucidating the progression from quiescent neural stem cells to the generation of diverse neural types, including Type 1 neural stem cells, Type 2A and 2B neural precursors, Type 3 neuroblasts, and their maturation into immature and eventually mature neurons. Circular arrows signify the proliferative potential at each stage, while straight arrows illustrate the directional transitions, with the capability of Type 1 cells to revert to quiescence. Additionally, the figure highlights the pivotal decision point where Type 2A precursor cells commit to either a neuronal or glial lineage (figure created with BioRender.com).

AHN is defined as the intricate process in which mature neurons are generated from human neural stem cells (hNSCs) in the adult central nervous system [11]. Studies conducted in human brain samples have established that new neurons are constantly produced in the subventricular zone (SVZ) of the lateral ventricles and sub-granular zone (SGZ) of the hippocampus throughout adult life (figure 1) [12–14]. Our current understanding suggests that mature interneurons originating from the SVZ integrate into the olfactory bulb, while granular neurons produced in the SGZ implicated in memory formation are incorporated into the neural circuits of the dentate gyrus [15,16]. The SVZ and SGZ both provide a permissive environment for NSCs via neurogenic niches—specialised microenvironments of supporting cells, signalling molecules, growth factors and neurotransmitters, ideal for neurogenesis [17,18].

Although the strict control and regulatory mechanisms of AHN are well-defined in murine models and primates, the regulatory processes in humans are yet to be fully elucidated due to evident biological, ethical and technical challenges [19,20]. Through the exploration of non-human and *in vitro* models, important signalling pathways governing AHN have been identified including the contributions of the canonical and non-canonical Wnt pathways, the Notch pathway, the bone morphogenic protein (BMP) pathway and the Sonic Hedgehog (Shh) pathway [21–24]. In efforts to unravel these in humans, researchers have developed various *in vitro* models employing hNSCs, human-induced pluripotent stem cells (iPSCs) and induced neurosphere (IN) cultures to examine these processes in the context of human biology as well as downstream applications including the discovery of novel molecular regulators of AHN and potential therapeutics.

AHN starts with the activation of quiescent NSCs—a specific subset of NSCs in a state of dormancy or a reversible cell cycle arrest often marked by ApoE3 expression and Notch pathway activation [25]. Subsequent phases then occur in a temporal manner: proliferation, determination of cell fate, differentiation, maturation, migration and integration of cells into existing neural networks [24]. These stages can be distinguished by specific neural markers (both at the protein and gene level) that fluctuate throughout each phase (figure 1). Activated NSCs (or Type 1 cells) are identified by neural markers such as glial fibrillary acidic protein (GFAP), Nestin (NES), SRY-box 2 (SOX2) and Musashi-1 (MSI1) [26]. Type 1 cells undergo slow asymmetric division to produce the highly proliferative Type 2 cells that continue to express NES [27–29]. Type 2 cells comprise two primary subtypes, Type 2A and Type 2B cells, that are further distinguished by the additional presence of PSA-NCAM with doublecortin (DCX) additionally expressed by only Type 2B cells. This is also the decision axis point at which Type 2 A cells commit to either a neuronal or glial fate, with a neuronal fate often identified by increased Achaete-scute homolog 1 (ASCL1) expression—a pro-activator blocking the return to quiescence [25,28]. These neuronal committed cells further differentiate into Type 3 neuroblasts expressing both DCX and PCA-NCAM as well as β-III tubulin (TUBB3) [30,31]. Immature neuroblasts eventually mature into neurons marked by Microtubule Associated Protein 2 (MAP2), Prox1 Homebox Transcription Factor (PROX1) and Neuronal Nuclei (Neun) [24].

The utilization of specific markers offers valuable insights into the distinct stages of neural cells during AHN. However, the optimal understanding is derived from the synergistic application of multiple markers and signalling pathways, including specific Wnt ligands [22,32]. Moreover, it is imperative to consider phenotypic changes, such as dendritic complexity, soma size, proliferation capacity, and if available, the spatial distribution within the hippocampus [27,29,33]. This integrative approach enhances the depth and precision of our comprehension of the intricate processes governing AHN, thereby contributing to the advancement of our knowledge in this field.

### 1.2. Current views on Alzheimer’s disease pathogenesis

#### 1.2.1. The amyloid cascade hypothesis

**Figure 2.**
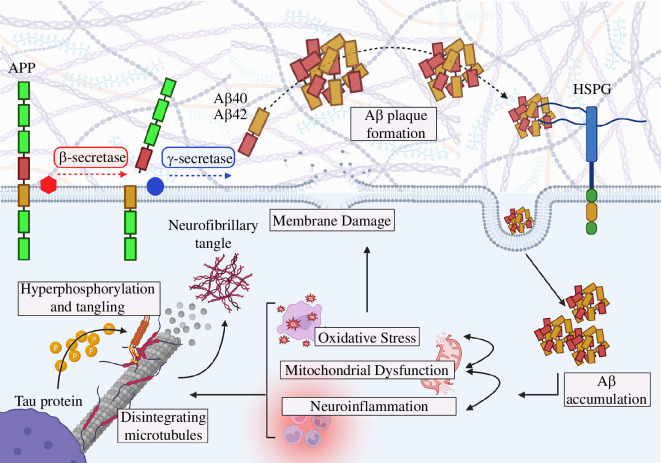
Pathogenesis of Alzheimer’s disease (AD). Impaired amyloid precursor protein (APP) processing pathway involves APP cleavage by β-secretase 1 (BACE1) and γ-secretase (PSENs) to produce toxic amyloid beta (Aβ40 and Aβ42) aggregates. Aβ forms the hallmark extracellular senile plaques ultimately internalized by transmembrane proteins such as the heparan sulfate proteoglycans (HSPGs). This contributes to oxidative stress, mitochondrial dysfunction and inflammation ultimately, leading to the hyperphosphorylation of tau protein (figure created with BioRender.com).

The amyloid cascade hypothesis is a widely accepted theory that proposes that the dysregulated processing of APP and the subsequent accumulation of amyloid beta (Aβ) plaques in the brain is the key event in the development of AD. According to this hypothesis, the overproduction and impaired clearance of Aβ leads to its accumulation resulting in neurotoxicity, synaptic dysfunction, oxidative stress and ultimately cognitive impairment [34]. Aβ is produced by impaired proteolytic cleavage of APP, mediated by β-secretase (BACE1) and γ-secretase (PSENs), resulting in the production of toxic oligomeric Aβ fragments (figure 2) [35]. The main isoforms of Aβ found in amyloid deposits are Aβ40 and Aβ42, consisting of 40 and 42 amino acids, respectively [36]. Aβ40 and Aβ42 concentrations are measured in cerebrospinal fluid with Aβ42/Aβ40 ratios of approximately 0.1 in non-AD populations which decreases to approximately 0.05 in AD patients [37]. Specifically, decreased Aβ42 has been shown to be associated with AD along with the deposition of compact Aβ plaques and congophilic amyloid angiopathy as shown in transgenic mouse models [38]. In healthy brains, the rates of Aβ synthesis and clearance are balanced, and Aβ fragments are cleared through various mechanisms, including via the actions of low-density lipoprotein receptor-related protein 1 (LRP1) and clusterin (CLU), or via enzymes such as neprylsin and matrix metalloproteinase-9 [39,40]. Despite extensive research dedicated to understanding the formation and regulation of APP and Aβ peptides, the limited success observed to date in clinical trials using anti-Aβ therapies suggests Aβ may not be the primary pathogenic factor in AD [7]. In contrast, the collective evidence suggests that while Aβ may contribute to the evolution of the disease, it is not the sole or primary factor driving AD progression [7].

More recently, research on APP processing has extended beyond AD and into the realm of neurogenesis. While APP is expressed in primary differentiated neuronal cells, early studies suggested that APP inhibition impedes neuronal adhesion, yet glial–glial cell adhesion remained unaffected [41]. However, the importance of the neural niche was also highlighted when embryonic *APP* knockout stem cells, displayed no significant effects on critical neuronal processes [42]. The narrative extends beyond APP, to the other peptides resulting from its processing, including soluble APP alpha (sAPPα), soluble APP beta (sAPPβ) and Aβ38,46,49—constituents of the stem cell niche that are often overlooked [36]. While a comprehensive understanding of the repercussions of most of these impairments remains elusive, sAPPα was found to enhance the proliferation of Type 3 neuroblasts, particularly in the SVZ—an area rich in sAPP binding sites *in vivo* [43]. This augmentation occurs through intensified epidermal growth factor-induced proliferation, a phenomenon confirmed *in vitro* in Type 2 murine INs [43]. This nuanced and understudied interplay among various APP-derived peptides underscores the complexity of their roles in neurogenesis and prompts further exploration into their specific and likely individual contributions in normal and pathogenic conditions.

#### 1.2.2. The tau hypothesis

The tau hypothesis proposes that the accumulation of hyperphosphorylated tau protein leads to the formation of neurofibrillary tangles (NFTs) (figure 2), suggesting it has a potential causative role in AD. This proposition gains significance in EOAD, where elevated tau levels are observed in the neocortical regions, sparing the lateral temporal regions including the hippocampus, in contrast to patients with LOAD [5,44]. Despite the absence of impact on the hippocampus, EOAD manifests AD and its hallmark cognitive symptoms, reinforcing the likelihood that tau pathologies contribute more substantially to the AD disease process.

Tau—encoded by microtubule-associated protein tau (*MAPT*)—is important for maintaining the structural integrity of neurons by stabilizing microtubules in axonic regions [45]. In AD, tau becomes hyperphosphorylated and accumulates in NFTs, a feature characterized by twisted fibrils known as paired helical filaments or straight filaments [46]. Initially, NFTs accumulate intraneuronally in the entorhinal cortex and are reported to appear approximately a decade prior to the appearance of Aβ pathologies [47–49]. Notably, these neurons reach deeper into the granular cell layer (GCL) of the dentate gyrus of the hippocampus (figure 1). While AHN is primarily restricted to the SGZ, newborn neurons migrate to the GCL in response to growth factors and molecular cues [33,50]. This is regulated by microtubules composed of α- and β-tubulin heterodimers and MAPs (including MAPT and MAP2) [51]. In traumatic brain injuries, this migration process is disrupted, causing neurons to prematurely relocate the GCL and undergo ectopic maturation, thereby influencing their morphological development [52]. Although the connection between impaired migration and the appearance of NFTs has not been explicitly examined, NFTs and hyperphosphorylated tau proteins have been identified in the brain as early as 6 h post-injury [53]. Remarkably, signs of impaired migration become evident 7 days after the injury, suggesting a relationship between tau pathologies and impaired AHN [52].

Numerous studies have also reported that neurodegeneration is primarily attributed to the spread of tau in the brain [54,55], with the correlation between tau and AHN offering a valid explanation. With the limited success of tau-targeting therapies in addressing AD, it is necessary to understand the temporal relationship between NFTs and impaired AHN [56]. Tau phosphorylation is important in healthy immature neurons primarily through glycogen synthase kinase-3 (GSK-3) [57]. GSK-3 is also a key participant in important neurogenic signalling pathways including the Wnt, Notch, BMP and Shh pathways; therefore, any dysregulation in these pathways may contribute to both hyperphosphorylation of tau and impaired AHN [58].

## 2. Impaired neurogenesis and Alzheimer’s disease

In exploring the temporal relationship of the hallmark AD amyloid and tau pathologies in the context of impaired AHN, it is essential to examine changes occurring in the early stages of AD that may influence the neurogenic niches. The Braak and Braak’s disease progression model provides a valuable staging system for this purpose [48]. This model offers an assessment of the severity of NFT pathology in the brain, identified through the examination of post-mortem brain samples [48]. In Braak stages I and II, NFTs predominantly localize within the entorhinal region, importantly sparing the neurogenic niches of the hippocampus. The hippocampus is affected when NFT progression advances to stages III and IV, with stages V and VI showing severe neocortical involvement [48]. Braak stages have been shown to be associated with AD severity and cognitive decline, with Braak stage II showing memory dysfunction but sparing executive, language or visuospatial domains and Braak stages V and VI indicating severe dementia [59].

In adhering to the central theme of this review, which focuses on impaired AHN *preceding* the appearance of NFTs, our assessment of current literature has focussed on the AD-induced changes identified in Braak stages I and II. These early stages offer a critical vantage point from which to understand the initial alterations occurring within neurogenic niches *before* the onset of pathological factors in the hippocampal regions.

As summarized earlier, the SVZ and SGZ emerge as conducive neurogenic microenvironments with impairments potentially contributing to memory deficits through compromised AHN. A study exploring post-mortem human brains showed increased MSI-1 expressing neural progenitor cells (NPCs) during Braak stage II [60]. This increased NPC proliferation was hypothesized to be a compensatory mechanism, possibly initiated by alterations in the entorhinal cortex, initially stimulating increased AHN and becoming dysfunctional after NFT deposition [60]. This was confirmed *in vitro* in a study exploring MSI-1 positive NPCs isolated from human post-mortem AD brain samples that still exhibited self-renewal capacity, yet reaching senescence earlier than cells from normal aged-matched controls [61]. Notably, this study did not concurrently explore NFT and amyloid pathologies which may have affected NPC dynamics. However, another study concluded that increased NPC proliferation did not correspond to enhanced AHN in pre-senile AD brain samples, instead attributing this to glial proliferation and vasculature associated changes [62]. It is important to highlight that this study only examined protein markers using immunohistochemistry (ki-67, DCX and GFAP), with no *in vitro* validation used to assess the proliferative potential of isolated NPCs, as in Lovell *et al.* [61]. Morphological analyses of GCL neurons also revealed substantial alterations, with clear reductions in soma size and the presence of two or more primary apical dendrites in Braak stages I/II patients [63]. Interestingly, the activation of GSK-3β mirrored the morphological changes observed in GSK-3β overexpressing murine models [64]. Recent evidence further substantiates these findings, demonstrating the loss of DCX positive immature neurons in Braak stage I AD brain samples when compared to healthy age-matched controls [65]. Importantly, this loss precedes the onset of clinical symptoms and the appearance of protein aggregates [65].

### 2.1. Potential links

#### 2.1.1. The Notch pathway

**Figure 3.**
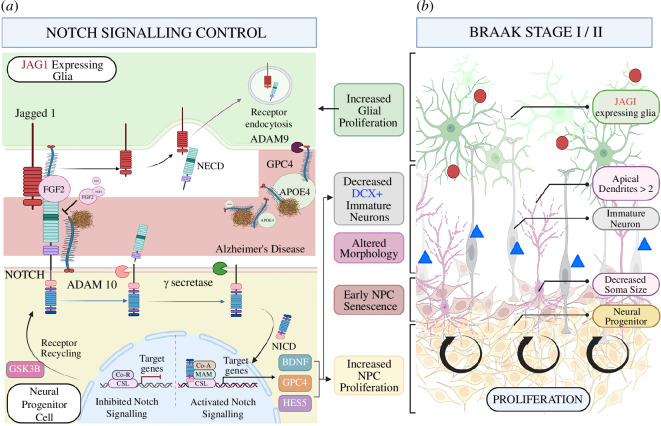
Contribution of the Notch signalling pathway to the changes in neurogenesis in the early AD stages. (*a*) The Notch pathway and its signalling cascade with both inhibited and activated Notch target genes. Theoretical changes in AD are shown in the left middle channel, showing increased GPC4 shedding by astrocytes ultimately increasing aberrant protein aggregations (both APOE and amyloid). This in turn inhibits FGF2-dependent Notch activation. (*b*) The neurogenic changes in the SGZ starting with increased NPC proliferation, decreased DCX+ (triangles) immature neurons and increased gliogenesis producing JAG+ (dots) glial cells (figure created with BioRender.com).

The Notch signalling pathway is crucial for the regulation of NSCs in the embryonic mammalian brain, balancing stem cell maintenance with daughter cell production and lineage fate [66]. Active Notch signalling serves to inhibit neuronal differentiation while promoting self-renewal and a glial fate in precursor cells [21]. Notch receptors are transmembrane heterodimers interacting with various ligands such as the Jagged (JAG1,2) and delta-like (DLL1,3,4) proteins in humans [67]. Upon ligand binding, a series of cleavage events involving γ-secretase (encoded by *PSEN1-2* among others) occur, leading to the release of the Notch intracellular domain (NICD) into the cytosol, with the extracellular domain endocytosed into the ligand-presenting cell (figure 3) [67]. The canonical Notch signalling pathway is characterized by the translocation of NICD to the nucleus, where it, in concert with mastermind-like proteins (MAML1–3), converts the recombining binding protein suppressor of hairless (RBPJ) complex from a transcriptional inhibitor to an activator, thereby regulating gene expression and neural fate via Hairy and Enhancer of split (HES1–5) transcription factors [68]. A significant portion of our understanding of the Notch signalling pathway in the nervous system has originated from studies conducted in *Drosophila* and *Xenopus*, with research now validated and extended to vertebrates, including mice and humans. While our knowledge has expanded to encompass these more complex organisms, there remains a need for further research to unravel the intricate mechanisms underlying this ancient and highly conserved signalling pathway.

In light of the consistent observations revealing increased proliferation of NPCs during the initial stages of AD, more detailed investigations into the Notch pathway for its involvement in these processes are warranted. Plausibly, the notable increase in NPC proliferation observed at Braak stage II may represent increased activation of the Notch pathway in response to the initial appearance of NFTs in the neurons branching from the entorhinal cortex to the dentate gyrus [60,69]. Alternatively, this may also occur via increased expression of Notch ligands, such as JAG1, shown to be predominantly expressed on GFAP positive astrocytic cells in the SVZ [70]. The increased glial proliferation observed in Braak stage I/II discussed earlier, could potentially increase JAG1 expressing cells and with it, Notch activation, further enhancing NPC proliferation. This has been demonstrated by the administration of the Notch ligand DLL4 to adult rats *in vivo*, resulting in increased NPC proliferation [21]. Increased Notch activation could also concurrently reduce neuronal differentiation, explaining the sharp drop in DCX-positive immature neurons in Braak stage I when compared to healthy controls [62,65]. Furthermore, post-mortem brain samples in the mild to moderate stages of AD have shown an increase of the downstream targets of Notch, such as HES5 and brain-derived neurotropic factor (BDNF), followed by a marked decline in later stages of the disease [71]. Interestingly, this pattern closely mirrors the observed oscillatory NPC proliferation in early (Braak stages I/II) to late (Braak stages III/IV) AD, as discussed earlier.

The contribution of the Notch pathway to AD onset gains credibility when exploring the early senescence displayed by AD patient-derived NPCs [61]. A study in human bone marrow-derived mesenchymal stem cells showed decreased Notch signalling linked to culture-induced cell ageing, a phenomenon successfully rescued by the activation of the Notch ligand Jagged 1-induced pathway [72]. This suggests that the early senescence of NPCs is reached through decreased Notch activation. The identity of molecular or other triggers for changes in Notch activation is not yet fully understood, however, the association between AD *PSEN* mutations, particularly L435F is noteworthy. This change-of-function mutation resulted in increased proliferation and depletion of post-mitotic neurons in hiPSC-derived cortical spheroids [73]. More recently, GSK-3β has emerged as an important regulator of the Notch pathway [74]. GSK-3β inhibition has been shown to increase Notch pathway activation mediated by enhanced Notch receptor recycling in tTA HeLa and U2OS cells. In contrast, GSK-3β activation resulted in dendritic tree morphological and synaptic density changes in GSK-3β overexpressing mouse models [64,74]. A potential mechanism for this could be that decreased Notch pathway activation results in early cell cycle exit of NSCs and the immature initiation of neurogenesis, resulting in the morphological changes observed in newborn neurons [66]. This is further validated by research showing changes in the Notch pathway in neuronal dysfunction and degeneration, with chronically decreased Notch signalling resulting in specific learning and memory deficits in *NOTCH^+/−^* and *RBPJ^+/−^* mouse models [75].

#### 2.1.2. Heparan sulfate proteoglycans

Discussion of stem cell maintenance and lineage specification necessitates consideration of heparan sulfate proteoglycans (HSPGs)—widely distributed glycoproteins facilitating crucial neurogenic cellular signalling through their interactions with the Wnt, BMP and Shh pathways [76]. HSPGs are composed of a core protein and one or more sulfated glycosaminoglycan (GAG) side chains that are synthesized in the Golgi apparatus via a complex post-translational biosynthesis process involving the sequential action of various glycosyltransferases and modification enzymes [77]. The resulting HS sulfation patterns determine the diverse interactions and functions of HSPGs, including their regulatory roles and interactions with AHN-relevant mediators including fibroblast growth factors (FGFs), BDNFs and platelet-derived growth factors [78]. Post-synthetically, modifications to HS structure and function occur enzymatically, through heparanase-mediated HS chain cleavage, shedding of HSPG core proteins by sheddases and 6-*O*-endosulfatase (Sulf1-2)-mediated sulfation changes [79].

While the exact mechanisms through which HSPGs may regulate the Notch signalling pathway are not fully understood, emerging evidence from non-human studies supports a link particularly through HS 3-*O* sulfation [80]. In neurons, the enzyme responsible for these motifs (HS 3-*O*-sulphotransferase 2 (HS3ST2)) was shown to be essential for the intracellular collection of HS and the subsequent induction of tau hyperphosphorylation and aggregation in zebrafish, human embryonic kidney (HEK293) and glioblastoma (SHSY5Y) cell models [81,82]. Interestingly, these intracellular HS depositions were shown to colocalize with tau proteins even prior to NFT formation [83,84]. While *HS3ST2* overexpression was shown to induce intracellular tau hyperphosphorylation, 6-*O* sulfation patterns were shown to be essential for extracellular tau binding and internalization, that was further supported by and observed decreased *SULF2* expression in AD brains [85,86]. In addition, evidence suggests a link for HSPG regulation of Notch signalling through FGF2 binding in embryonic day 10 neuroepithelial precursors, inhibiting neural differentiation and promoting proliferation [87]. While FGF2 and HS interactions are known to occur primarily through 2-*O* sulfation patterns, HS also interacts with senile plaques by competitively binding to the same 2-*O*-sulfated iduronic acid residues [88–90]. This competitive binding may decrease FGF2 and Notch interactions in the later stages of AD resulting in impaired neuronal differentiation [87].

These studies underscore the crucial role of HS and its sulfation patterns in the initiation and progression of AD. HS influences the induction of tau hyperphosphorylation, aggregation, Aβ entry into cells, and has the potential to dysregulate the Notch pathway, contributing to impaired neurogenesis [91]. Most recently, it was shown that ApoE4 also exhibits a threefold higher binding affinity for HSPGs than the neutral ApoE3 and protective ApoE2 isoforms [92]. While this study did not explore specific HSPG subtypes, Saroja *et al*. suggested that ApoE4 exhibits a higher binding affinity for glypican 4 (GPC4)—a member of transmembrane HSPG family [93]. GPC4 has been shown to be highly expressed in NPCs and in APOE4-carrying post-mortem AD brains, as such, the increase in NPCs observed at early-stage AD may contribute to the progression of AD through ApoE4 mediated tau hyperphosphorylation in high risk ApoE4 carriers [93,94]. This effect could be further intensified through astrocytic GPC4 secretions facilitated by proteolytic shedding [95]. Additionally, the elevated number of NPCs expressing higher levels of GPC4 likely adds to the amplification of this phenomenon [94].

### 2.2. Our hypothesis: Notch, HSPG and impaired neurogenesis initiate AD

**Figure 4.**
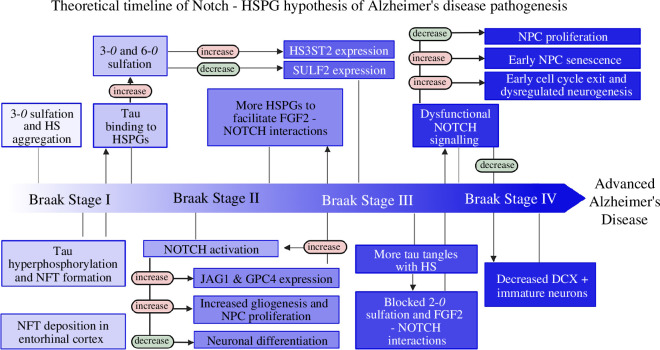
Proposed new hypothesis for the early aetiology of Alzheimer’s disease. This hypothesis integrates Notch signalling and heparan sulfate proteoglycans (HSPGs) in impaired adult hippocampal neurogenesis (AHN) as an early event in AD. HS—Heparan Sulfate, NFT—Neurofibrillary Tangles, NPC—Neural Progenitor Cells, GPC4—Glypican 4, JAG1—Jagged 1, DCX—Doublecortin, FGF2—Fibroblast Growth Factor 2, HS3ST2—Heparan Sulfate Glucosamine 3-O-Sulfotransferase 2, SULF2—Sulfatase 2.

When examining the intricate interplay of molecular events underlying AD, a novel hypothesis emerges, weaving together the roles of Notch signalling and HSPGs in impaired AHN as an early event of AD pathogenesis (figure 4). The ageing brain accumulates HS within cells, due to natural changes in sulfation patterns and quantities of GAG side chains, as demonstrated in rat models [96]. Further contributing factors may include the ApoE4 haplotypes and potentially the common aged care anticoagulant, heparin, an HS analogue, known for its high 3-*O* sulfation [97,98]. HS accumulation along with high 3-*O* sulfation triggers a cascade of events: intracellular tau hyperphosphorylation and aggregation, accompanied by overactive Notch signalling initiated in the entorhinal cortex as seen in Braak stage I [48]. This synergistic effect leads to the over-proliferation of NPCs and/or glial cells as observed in previous studies [60,62], creating a positive feedback loop that further amplifies Notch activation through increased glial JAG1 expression [70]. Concurrently, GPC4 shedding from astrocytes and increased NPC GPC4 expression provide a permissive environment for ApoE4 and intensified tau hyperphosphorylation [93–95]. This intricate dance continues as tau traps APP endosomes [47], triggering Aβ formation, exacerbated by the increased availability of HSPG 6-*O* sulfation—essential for tau binding and further hindering BACE1 activity [86,90]. However, the arrival of toxic Aβ introduces a counterforce, competing for 2-*O* sulfation, ultimately blocking FGF2-induced Notch activation [87]. This dysregulation culminates in aberrant neurogenesis, marked by peculiar neural morphologies and, ultimately, cognitive decline.

## 3. Future directions

In the realm of AD research, the predominant focus on the amyloid and tau hypotheses may have obscured research into alternative potential initiating factors. Further exploration of the alterations in HS sulfation patterns and quantity in human models offers a unique and clinically relevant perspective to better explore the pre-NFT aggregation phase, particularly in specialized regions such as the entorhinal cortex as per the Braak model [48]. Investigating HS aggregation and aberrant sulfation patterns may provide promising AD therapeutic targets within sulfation enzymes to help further unravel the temporal relationship between HS changes and tau hyperphosphorylation. Amid the debate over whether tau or amyloid pathologies come first, emphasizing the need to delve into what precedes *both* of these events could unveil novel participants, such as markers within the Notch signalling pathway (linked to APP processing via common enzymes like γ-secretase) and HSPGs (linked to both pathologies through distinct sulfation patterns). Given the potential role of Notch signalling in regulating aberrant AHN as observed in early AD aetiology, further studies should scrutinize the intricate mechanisms connecting HSPGs to Notch signalling. With their importance in Drosophila Notch pathway activation already established, exploration of 3-*O* sulfation pattern gains will provide valuable insight into the regulation of AHN [80]. The use of heparin, an HS analogue with high 3-*O* sulfation patterns in aged care, warrants further clinical consideration regarding its contribution to early HS aggregation and its impact on the Notch pathway, another direction for future research [99]. Interestingly, the addition of heparin to many *in vitro* models may already provide the foundation for a better understanding of these interactions and their impact on AD pathology [45,78,86,100–102].

In summary, this review propels a paradigm shift in AD research, suggesting unexplored correlations between AHN, Notch and HSPGs as key participants in AD initiation and progression. While not an exhaustive meta-analysis, this new perspective challenges the dependence on amyloid and tau as triggers of AD and encourages their consideration as symptoms only in AD research.

## Data Availability

This article has no additional data.
